# The Value of Continuity between Primary Care and Surgical Care in Colon Cancer

**DOI:** 10.1371/journal.pone.0155789

**Published:** 2016-05-24

**Authors:** Tanvir Hussain, Hsien-Yen Chang, Ngoc-Phuong Luu, Craig Evan Pollack

**Affiliations:** 1 Department of Medicine, Division of General Internal Medicine, University of Nebraska Medical Center, Omaha, Nebraska, United States of America; 2 Department of Health Policy & Management, Johns Hopkins Bloomberg School of Public Health, Baltimore, Maryland, United States of America; 3 Department of Medicine, Division of General Internal Medicine, Johns Hopkins University School of Medicine, Baltimore, Maryland, United States of America; University of Louisville School of Medicine, UNITED STATES

## Abstract

**Background:**

Improving continuity between primary care and cancer care is critical for improving cancer outcomes and curbing cancer costs. A dimension of continuity, we investigated how regularly patients receive their primary care and surgical care for colon cancer from the same hospital and whether this affects mortality and costs.

**Methods:**

Using Surveillance, Epidemiology, and End Results Program Registry (SEER)-Medicare data, we performed a retrospective cohort study of stage I-III colon cancer patients diagnosed between 2000 and 2009. There were 23,305 stage I-III colon cancer patients who received primary care in the year prior to diagnosis and underwent operative care for colon cancer. Patients were assigned to the hospital where they had their surgery and to their primary care provider’s main hospital, and then classified according to whether these two hospitals were same or different. Outcomes examined were hazards for all-cause mortality, subhazard for colon cancer specific mortality, and generalized linear estimate for costs at 12 months, from propensity score matched models.

**Results:**

Fifty-two percent of stage I-III colon patients received primary care and surgical care from the same hospital. Primary care and surgical care from the same hospital was not associated with reduced all-cause or colon cancer specific mortality, but was associated with lower inpatient, outpatient, and total costs of care. Total cost difference was $8,836 (95% CI $2,746–$14,577), a 20% reduction in total median cost of care at 12 months.

**Conclusions:**

Receiving primary care and surgical care at the same hospital, compared to different hospitals, was associated with lower costs but still similar survival among stage I-III colon cancer patients. Nonetheless, health care policy which encourages further integration between primary care and cancer care in order to improve outcomes and decrease costs will need to address the significant proportion of patients receiving health care across more than one hospital.

## Introduction

Improving care transitions and continuity between primary care and specialty care are a focus of health care reform and important for achieving high value cancer care—that is, care which achieves optimal patient outcomes while containing costs [1–3]. Primary care providers (PCPs) are routinely responsible for cancer screening; investigating symptoms that reveal a cancer diagnosis; initiating referrals for oncologic treatment; managing comorbidities for chronically ill patients; and providing patient-centered assessments for the role of aggressive therapy when the role of treatment is uncertain, such as adjuvant chemotherapy in stage II colon cancer [4–7]. PCPs also assist with cancer surveillance during survivorship [4, 5]. Therefore, improving continuity in the transitions between primary care and cancer care may lead to higher quality and more efficient care [8].

One factor that may be associated with improved continuity between primary care and cancer care is whether these providers are part of the same hospital system. Cancer providers who are affiliated with the same hospital and work together more frequently may face fewer barriers to coordinating care [9, 10]. Patients receiving treatment for other conditions, such as chronic diseases, from more than one hospital have poorer outcomes, possibly due to worse access to patient data and communication challenges between co-managing providers [11–14]. To our knowledge, the association between patients receiving primary care and cancer care from physicians affiliated with the same hospital and mortality or costs has not been evaluated.

We focus on colorectal cancer, the third leading cause of cancer mortality and second most costly cancer in the U.S. population [15]. Guidelines for the medical care of stages I through III colon cancer recommend timely referral to surgery after diagnosis to improve survival [7]. Therefore, effective collaboration between primary care and surgical care is integral across these three stages of colon cancer [16–19]. Regardless of the diagnosing physician, nearly half of colorectal cancer patients see their PCP between diagnosis and surgery [20]. Further, use of primary care increases in the first year after colorectal cancer diagnosis—most commonly for evaluation and treatment of cancer-related complications and mood disorders [21]. Half of physician visits in the year following colorectal cancer diagnosis are with PCPs [22]. With one-third of colon cancer patients experiencing recurrence and a survival less than two years, close follow-up and coordination of care with an established PCP after resection of colon cancer may improve outcomes and decrease cost [23, 24]. However, inadequate communication between PCPs and surgeons is still prevalent [25]. Fewer barriers to continuity and coordination of care may exist if patients receive both their primary care and surgical care from physicians affiliated with the same hospital.

Using national cancer registry and administrative claims data from the U.S., we investigated whether stage I through III colon cancer patients who receive surgical care within the same hospital where their PCP mainly practices have improved overall survival, colon cancer specific survival, and lower twelve-month costs of care compared to patients who receive their surgical care from a different hospital.

## Methods

### Study Population

Using methodology described previously [9, 10], from the Surveillance, Epidemiology, and End Results Program Registry (SEER)-Medicare files we identified patients with colon cancer diagnosed between 2000 and 2009. SEER-Medicare files link data from the SEER population-based cancer registry, encompassing approximately 28% of the US population, to Medicare claims data; complete claims data is available for approximately 93% of the patients with Medicare in SEER [26].

**Fig 1:**
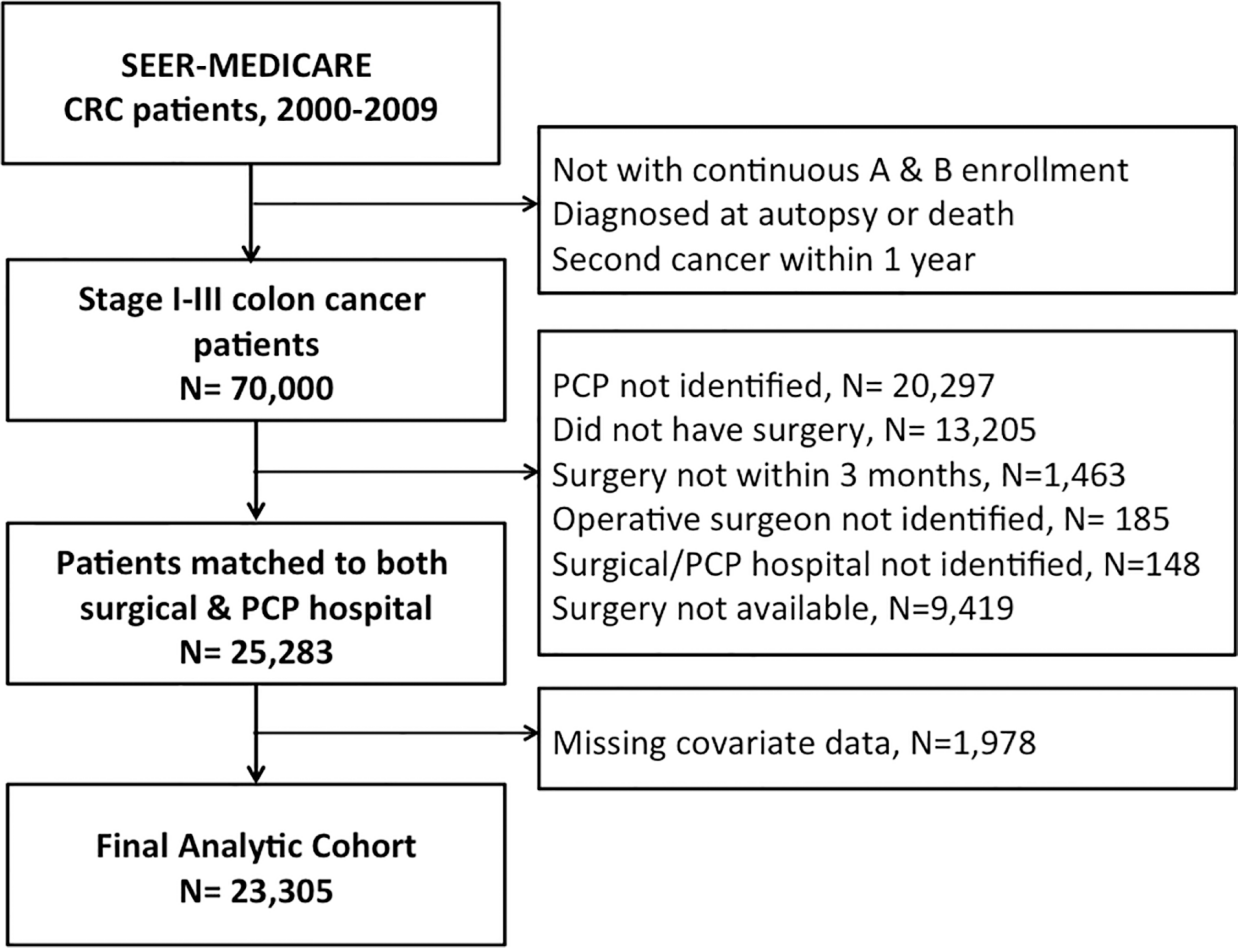
Flow chart of inclusions & exclusions for analytic cohort.

For reasons similar to those detailed previously [9, 10], inclusion and exclusion criteria are as follows. We included only those patients with stage I through III colon cancer with continuous Part A and B Medicare coverage during the 12 months before and 12 months after their diagnosis date (Fig 1). Patients were excluded for the following reasons: if younger than 66 at diagnosis (as Medicare eligibility, and thus claims data, routinely becomes available at age 65); if enrolled in a health maintenance organization, HMO, during the two-year interval (in which case additional claims would exist outside of Medicare); if colon cancer was diagnosed at autopsy or death; or if diagnosed with a second cancer within twelve months of colon cancer diagnosis. We excluded patients who did not have a primary care visit in the twelve months prior to diagnosis (n = 20,297); did not undergo any surgery for colon cancer (n = 13,205) or underwent colon cancer surgery beyond three months following their diagnosis (n = 1,463); did not have an identifiable surgeon providing operative care (n = 185); and could not be assigned to a hospital for either their primary care or surgical care (see below, n = 148). Because we were interested in patients who could potentially receive their surgical care at the same hospital as the one their PCP was assigned to, we excluded patients whose PCP-assigned hospital (see below) did not appear to offer surgical care to colon cancer patients in our cohort (n = 9,419). The mechanism for missingness of covariate data appeared to be completely at random; therefore, we further excluded those who had missing covariate information (n = 1,978). The final analytic cohort consisted of 23,305 patients.

### Measures

#### Outcomes

The primary outcomes were all-cause mortality and total cost of care at 12 months after diagnosis. Survival time spanned from the date of colon cancer diagnosis to Medicare date of death, or a censor date of December 31, 2011. We calculated costs as the total reimbursement made on patient claims—rather than the total charges, which can vary significantly between providers and institutions—using the Medicare Provider Analysis and Review File, Carrier Claims, and the Outpatient Statistical Analysis File [27]. Colon cancer specific mortality was a secondary outcome. The censor date for colon cancer specific mortality was December 31, 2009; cause of death was not available after this date.

#### Provider and hospital assignment

Comparable to published methods for provider and hospital assignment [9, 10], the operative surgeon and the hospital where the surgery was performed were identified using SEER-Medicare billing codes. See S1 Table for list of surgical ICD-9 codes used. For primary care, we assigned patients to the PCP—defined as general practitioners, family practitioners, geriatricians, and internal medicine physicians without subspecialty training—who billed for the plurality of the patient’s primary care visits in the year prior to diagnosis [28, 29]. Identifying the PCP who provided care in the year *prior* to diagnosis, rather than the year of diagnosis, has been used in previous studies and associated with cancer outcomes [30, 31]. PCPs were assigned to the hospital where they were most likely to practice following the approach of Bynum and colleagues [32]. Specifically, PCPs were assigned to the hospital in which they billed for the most inpatient care. For PCPs who did not bill for any inpatient care, they were assigned based on where the plurality of their patients was admitted. To increase the accuracy of the PCP’s hospital assignment, in addition to considering the hospital billing and admissions of their cancer patients, we additionally used the 5% Medicare sample of non-cancer patients. We linked these non-cancer patients to their plurality PCPs and their PCPs to hospitals, using the same algorithm. Thus, the PCP’s hospital assignment was based on the plurality of the PCP’s inpatient billings/admissions for cancer and non-cancer patients.

Patients were classified as receiving primary care and surgical care from the same hospital if they underwent their operation at the same hospital where their PCP was assigned.

#### Covariates

We included covariates in our analysis that have been used in similar studies previously [9, 10] and are available in the SEER-Medicare dataset. Patient level covariates included age, gender, Medicare self-reported race (Black, White, other), census tract median household income (in quartiles), year of colon cancer diagnosis, Charlson comorbidity score for the 12 months prior to diagnosis, urban/rural residence, and the SEER geographic region (site) in which the patient resides. Cancer characteristics included tumor grade, adequate lymph node resection during surgery (≥12 lymph nodes), and cancer substage, which became available in 2004 [33]. Physician level covariates include yearly surgical volume, which was calculated from the total number of all colon cancer patients (stages I-IV) on whom surgeons operated in a given year [34], and modeled in quartiles (<2, 2, 3–4, ≥5 cases/year). Hospital level covariates included National Cancer Institute (NCI)-recognized status of the surgical hospital, academic hospital status (whether a teaching hospital or affiliated with one) and for-profit status (for-profit or government/voluntary non-profit) for both surgical and PCP hospital. We tabulated the total volume of patients who underwent colon resection at each surgical hospital, which we categorized in quartiles (0–129, 130–210, 211–320, >321 cases) [35, 36].

### Analysis

We performed multivariable logistic regression controlling for those patient, provider, and hospital characteristics used in previous studies [9, 10] to identify characteristics associated with patients receiving their primary care and surgical care at the same or different hospital(s). We modeled all-cause mortality using Cox proportional hazards and estimated colon cancer specific mortality using Fine and Gray’s method for competing risk subhazards, where death from other causes was a competing risk [37]. We used the Grambsch and Therneau test of non-zero slope, to assess the proportional hazards assumption throughout the follow-up period [38].

The difference in costs of care at twelve months was modeled with generalized linear models [39]. The modified Park test guided selection of the distribution and link functions for the generalized linear models [40]; we used a gamma variance distribution and log link to model cost data. We accounted for inflation over time. Using the annual Gross Domestic Product price index, all cost data were inflated to dollar values in 2009 [41].

Similar to prior published approaches [9, 10], we used propensity-matched doubly robust regression models to estimate both survival and costs. Propensity score models included all patient, physician, and hospital characteristics and were calculated using psmatch2 version 3.0 in STATA [42]. Variance inflation factors were examined to check for multi-collinearity between provider and hospital characteristics before inclusion in the propensity score models. Nearest neighbor 1:1 matching with caliper of 0.01 with no replacement optimized balance of the data, and was used for all outcome regression models. To correct for clustering within each PCP-surgical hospital pair, generalized estimating equations (GEE) for cost models and robust variance estimation for survival analysis were used.

To check for robustness of our main findings, we performed several sensitivity analyses. First, we stratified all our analyses by cancer stage. Second, we modeled analyses controlling for substage among patients diagnosed in 2004 onward, as cancer substage data became available in 2004. Third, we included those patients who had missing covariate data by using multiple imputation to model missing values based on all other available patient information. Fourth, we examined total cost of care at six months for our analytic cohort, and costs at both six and twelve months only amongst those colon cancer patients who survived six and twelve months after diagnosis respectively. Fifth, physician and hospital characteristics may have been unknown to many patients prior to receiving their care, thereby not affecting their location of primary care or surgical care; therefore, we re-ran our propensity models including only patient characteristics. Sixth, because there are important documented socioeconomic disparities in colon cancer care and outcomes, we tested for potential interactions between one versus two hospital care with race/ethnicity, median census tract income, and urban/rural residence.

In our final sensitivity analysis, among stage III patients who received medical oncologic care, we identified their primary medical oncologist in the year following their diagnosis and assigned each medical oncologist to a hospital, similar to the algorithm used PCP-hospital assignment and according to methods published prior [9, 10]. In our prior work, we found that cost of care for stage III colon cancer patients varied depending on whether their surgeon and medical oncologist were affiliated with the same or different hospital(s) [9]. We therefore examined how the number of different hospitals involved in a patient’s care—one hospital representing patients whose primary, surgical, and medical oncologic care were all assigned to one hospital, and three hospitals representing primary, surgical, and medical oncologic care were all assigned to different hospitals—is associated with survival and cost.

All analyses were completed with STATA IC 12.1. Data used in this study were de-identified, and considered a limited data set, which requires that investigators sign a Data Use Agreement with SEER-Medicare identifying the specific analyses that will performed and the investigators who will use the data, prior to receiving the data. This exception allows for the release of the SEER-Medicare data without obtaining authorization from individual patients [43]. Further, our study received approval from the Johns Hopkins University School of Medicine Institutional Review Board.

## Results

In our cohort of 23,305 stages I through III colon cancer patients, 52.2% of patients received their primary care and surgical care from the same hospital—that is, their PCP was primarily affiliated with the same hospital where they received operative care (Table 1). In adjusted analyses, patients receiving their primary and surgical care from the same hospital were less likely to undergo surgery with the lowest volume surgeons and were less likely to undergo their surgery at a NCI-designated cancer center compared those receiving care from different hospitals.

**Table 1:** Characteristics colon cancer patients, by receipt of same versus different hospital primary care and surgical care.

	Same Hospital Care Delivery	Different Hospital Care Delivery	p-value	Odds Ratio (95%CI) for Same Hospital Care Delivery*
	n = 12,156 (52.2%)	n = 11,149 (47.8%)		
**PATIENT-LEVEL CHARACTERISTICS**				
**Age**			<0.001	
>65–70	1610 (13.2)	1512 (13.6)		Ref
71–75	2434 (20.0)	2479 (22.2)		0.96 (0.90–1.01)
76–80	2978 (24.5)	2741 (24.6)		0.99 (0.91–1.10)
81–85	2797 (23.0)	2446 (21.9)		1.06 (0.96–1.17)
>86	2337 (19.2)	1971 (17.7)		1.05 (0.97–1.16)
**Female**	7179 (59.1)	6513 (58.4)	0.32	0.99 (0.94–1.05)
**Race**			<0.001	
White	10828 (89.1)	9557 (85.7)		Ref
Black	716 (5.9)	800 (7.2)		0.96 (0.78–1.19)
Other	712 (5.0)	792 (7.1)		1.08 (0.80–1.57)
**Census Tract Median Income**			0.01	
Lowest Quartile	2964 (24.4)	2853 (25.6)		Ref
2^nd^ Quartile	3069 (25.3)	2764 (24.8)		1.03 (0.88–1.21)
3^rd^ Quartile	3131 (25.8)	2696 (24.2)		1.05 (0.87–1.27)
Highest Quartile	2992 (24.6)	2836 (25.2)		1.02 (0.80–1.32)
**Urban/Rural Residence**			<0.001	
≥1 million population	6168 (50.7)	6479 (58.1)		Ref
≥250000 to < 1 mil	3992 (32.3)	3006 (27.0)		1.12 (0.80–1.56)
<250,000	2066 (17.0)	1664 (14.9)		1.02 (0.71–1.49)
**Charlson Comorbidity Score**			0.11	
0	6755 (55.6)	6042 (54.2)		Ref
1	3092 (25.4)	2921 (26.2)		0.95 (0.89–1.01)
≥2	2309 (19.0)	2186 (19.6)		0.97 (0.90–1.06)
**Stage**			0.008	
I	1447 (11.9)	1185 (10.6)		Ref
II	8353 (68.7)	7798 (69.9)		0.89 (0.77–1.03)
III	2357 (19.4)	2167 (19.4)		0.91 (0.76–1.08)
**Substage**^†^				
Stage II			0.98	N/A^‡^
IIa	2334 (89.0)	2332 (89.0)		
IIb	289 (11.0)	287 (11.0)		
Stage III			0.71	N/A^‡^
IIIa	239 (11.2)	240 (11.1)		
IIIb	1226 (57.8)	1226 (56.7)		
IIIc	658 (31.0)	695 (32.2)		
**Tumor Grade**			0.008	
Well Differentiated	1280 (10.5)	1025 (9.2)		Ref
Moderately	8353 (68.7)	7798 (69.9)		0.89 (0.77–1.03)
Poorly	2356 (19.4)	2166 (19.4)		0.91 (0.76–1.08)
Undifferentiated	167 (1.3)	160 (1.4)		0.79 (0.59–1.06)
**Adequate lymph node resection**			0.27	
<12	4623 (38.0)	4162 (37.3)		Ref
≥12	7533 (62.0)	6987 (62.7)		1.02 (0.93–1.13)
**PROVIDER-LEVEL CHARACTERISTICS**				
**Yearly surgical volume**			<0.001	
Lowest Quartile	2187 (18.0)	2637 (23.7)		Ref
2^nd^ Quartile	2381 (19.6)	2169 (19.5)		1.32 (1.20–1.47)
3^rd^ Quartile	3818 (31.4)	3068 (27.5)		1.43 (1.30–1.59)
Highest Quartile	3770 (31.0)	3275 (29.4)		1.52 (1.35–1.61)
**HOSPITAL-LEVEL CHARACTERISTICS**				
**Surgical Hospital**^§^ **Volume**			<0.001	
Lowest Quartile	2554 (21.1)	3204 (29.0)		Ref
2^nd^ Quartile	3215 (26.5)	2541 (23.0)		1.19 (0.89–1.75)
3^rd^ Quartile	3163 (26.1)	2759 (24.9)		1.17 (0.83–1.64)
Highest Quartile	3181 (26.3)	2561 (23.1)		1.25 (0.83–1.88)
**NCI Cancer Center**				
Surgical Hospital^§^	200 (1.7)	516 (4.6)	<0.001	0.30 (0.15–0.60)^¶^
**Academic Center**				
Surgical Hospital^§^	6551 (53.9)	5831 (52.3)	0.02	0.83 (0.65–1.06)^#^
PCP Hospital^‖^	6551 (53.9)	6123 (54.9)	0.15	0.92 (0.92–1.13)^#^
**For Profit Hospital**				
Surgical Hospital^§^	813 (6.7)	1160 (10.4)	<0.001	0.83 (0.61–1.12)**
PCP Hospital^‖^	813 (6.7)	1027 (9.2)	<0.001	1.06 (0.78–1.43)**
**OUTCOME MEASURES**				
**Number of Deaths**	6150 (50.6)	5238 (47.0)	0.041	N/A
**Median total cost for first year of care (IQR)**	$44,722 ($30,432-$75,219)	$50,707 ($22,930-$81,653)	<0.001	N/A

*: Odds Ratios are fully adjusted for all other variables listed here (except substage as this is not available until 2004), as well as diagnosis year and SEER site which are not shown

†: Sample restricted to those diagnosed between 2004 onward, for whom this data are available

‡: Not included in model because substage data is available only for all patients, only after 2004

§: The hospital where the patient had surgery

‖: The hospital with which the patient’s PCP is primarily affiliated

¶: Reference group includes hospitals not designated as NCI centers

#: Reference group includes non-academic hospitals.

**: Reference group includes non-profit hospitals.

**Fig 2:**
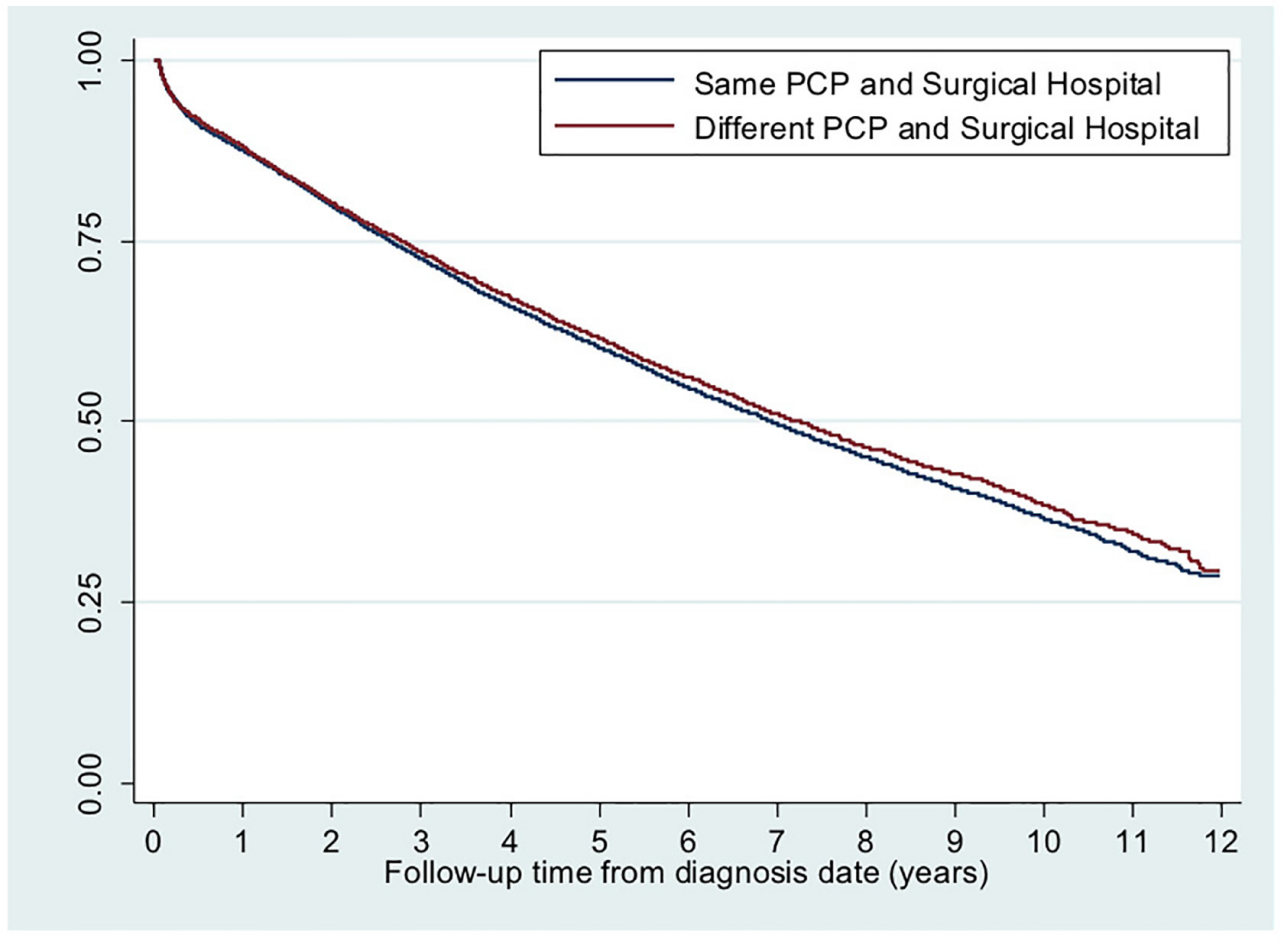
Kaplan Meier survival curve for all-cause mortality by same versus different hospital primary and surgical care delivery. Average follow-up time: 4.8 years. Total follow-up time: 112,820 years. Log rank survival function: chi square = 0.22, p = 0.52. Test of non-zero slope: p = 0.31.

**Fig 3:**
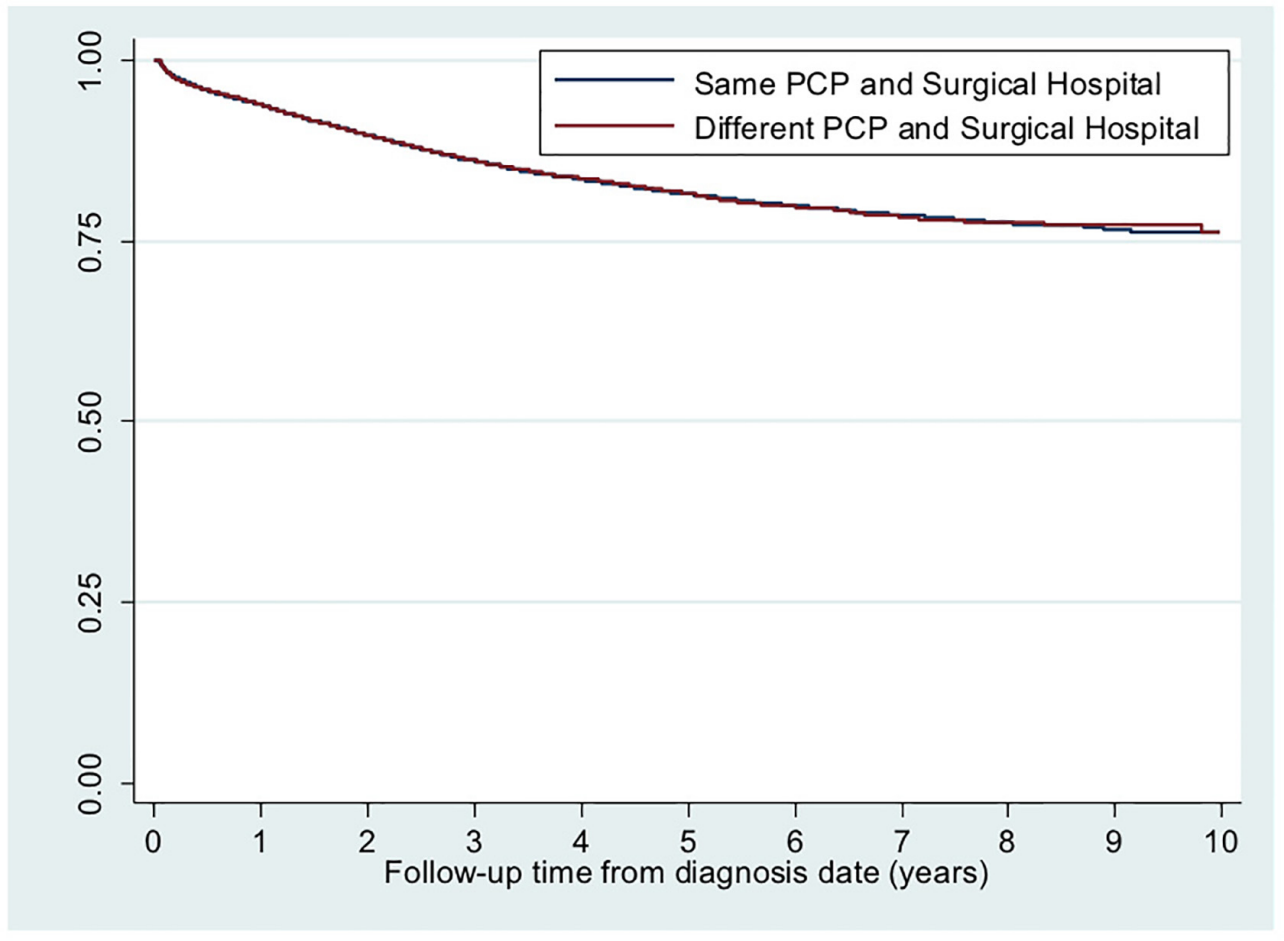
Kaplan Meier survival curve for colon cancer specific mortality by same versus different hospital primary and surgical care delivery. Average follow-up time: 3.7 years. Total follow-up time: 86,567 years. Log rank survival function: chi square = 0.10, p = 0.75. Test of non-zero slope: p = 0.21.

As shown in Figs 2 and 3, there were no significant differences in all-cause mortality or colon cancer specific mortality between patients receiving primary care and surgical care for colon cancer at same versus different hospital(s). Mean unadjusted cost of care at 12 months was approximately $6,000 lower for patients receiving same hospital care ($44,722 versus $50,707, p<0.001).

Propensity score matching procedures led to substantial improvement and optimal balance across covariates (S2 Table). Mean bias was less than 5% across nearly all covariates and overall mean bias for all covariates decreased from 5.5 to 1.4 after propensity score matching. The propensity score-matched doubly robust Cox proportional hazards regression for all-cause mortality, competing risks subhazard regression for colon cancer specific mortality, and total cost of care at 12 months for all stages combined and stratified by stage are found in Table 2.

**Table 2:** All-cause & colon cancer specific mortality and cost at 12 months associated with receiving same versus different hospital primary care and surgical care from propensity score-matched doubly robust models*.

	Hazard Ratio for All-Cause Mortality	Subhazard Ratio for Colon Cancer Specific Mortality	Dollars Saved at 12 months from Generalized Linear Model Estimates
	(HR, 95%CI)	(SHR, 95% CI)	(Dollars, 95% CI)
**Stages I-III (n = 23,305)**			
**Same Hospital**	Ref	Ref	Ref
**Different Hospital**	1.04 (0.99–1.09)	1.02 (0.97–1.06)	$8,836 ($2,746-$14,577)
**Stage I (n = 2,632)**			
**Same Hospital**	Ref	Ref	Ref
**Different Hospital**	0.99 (0.94–1.04)	1.01 (0.93–1.06)	$2,841 ($523-$5683)
**Stages II (n = 16,151)**			
**Same Hospital**	Ref	Ref	Ref
**Different Hospital**	1.02 (0.96–1.07)	1.03 (0.91–1.11)	$13,046 ($3,228-$22,326)
**Stages III (n = 4,522)**			
**Same Hospital**	Ref	Ref	Ref
**Different Hospital**	0.97 (0.92–1.05)	0.98 (0.89–1.07)	$22,197 ($5,778-$41,088)

*: Estimates are fully adjusted for and have been matched on all patient, provider, and hospital characteristics listed in Table 2, as well as diagnosis year and SEER region.

Receiving primary and surgical care from the same hospital compared to receiving care at different hospitals was not associated with all-cause (Hazard Ratio 1.04, 95%CI 0.99–1.09) or colon cancer specific mortality (Subhazard Ratio 1.02, 95%CI 0.97–1.06) when considering all stages together, or for each stage separately. However, patients who received primary and surgical care from the same hospital had significantly lower total costs than those who received care at different hospitals ($8,836, 95% CI $2,746-$14,577). We observed statistically significant savings both in outpatient ($5,991, 95% CI $1,198 to $9,986) and inpatient care ($1,625, 95% CI $925 to $2,250), but not provider billings for patients who received care from the same hospital. When examining costs by stage of colon cancer, the cost difference between those receiving primary and surgical care at the same hospital compared to different hospitals became larger with higher stage cancers: from $2,841 (95% CI $523-$5,683) for stage I patients to $22,197 ($5,778-$41,088) for stage III patients. Our main findings did not significantly change in any of the sensitivity analyses (S3 Table), and all pre-specified tests of statistical interactions terms were also statistically non-significant.

Among stage III patients, the more hospitals involved in a patient’s care, the more costly the care without differences in survival (S3 Table). Costs for those receiving all their care (primary, surgical, and medical oncologic care) at the same hospital were $19,297 lower (95% CI $16,013-$24,765) than those receiving care at two different hospitals and $25,973 lower (95%CI $20,843-$31,114) than those receiving care at three different hospitals.

## Discussion

Half of stage I through stage III colon cancer patients received care at different hospitals during their transition from primary care to surgical cancer care. While we did not observe differences in either all-cause or colon cancer specific mortality, we did find significant differences in the twelve-month cost of care—on average, 20% lower median cost at twelve months among patients who received their primary and surgical care at the same hospital. These results raise important implications for attempts to improve continuity during transitions between primary care and cancer care, particularly in the setting of current health care reforms that seek to develop more integrated delivery models for patients with complex illness.

A considerable proportion of patients switched from their routine primary care setting to another hospital for their surgical cancer care, even though surgical care was available at the PCP’s assigned hospital. Patient comorbidities and severity of cancer stage and grade did not appear to affect whether patients sought surgical care outside their location of primary care. Instead, characteristics of the operating surgeon (volume) and the surgical hospital (NCI status) were associated with patients receiving their surgical care at a hospital different from their primary care. With less than 70 NCI cancer centers, patients are likely required to travel to one of these NCI centers [44] which have been shown to have improved patient outcomes [45]. In contrast, patients who stayed in the same hospital were less likely to receive care from a low volume surgeon. It is unclear why patients who sought surgical care at a different hospital were more likely to undergo surgery by a lower volume surgeon; perhaps patients expected shorter delays to surgery. In exploratory analyses, we found that the time to surgery was similar in both groups (approximately 20 days).

We did not find differences in overall or colon cancer specific mortality between those who received both primary care and surgical care at the same versus different hospital(s). Although colon cancer patients with higher utilization of primary care prior to diagnosis have lower colon cancer specific and all-cause mortality [30], our study suggests that the location of their primary care, relative to their surgical cancer care, is not linked with survival. It is possible that patient-reported outcomes such as patient experience and quality of life may vary between the two groups, as primary care physicians who practice in similar institutions as surgeons may be able to play a more active role in advocating for patient goals and managing depression and pain during the acute phase of cancer treatment [6, 46, 47]. On the whole, this may suggest that patients who remain at the same institution for surgical care are not receiving poorer quality care. These results build on our prior study which showed no difference in survival for stage III colon cancer patients based on whether surgeons and medical oncologists were affiliated with the same hospital [9]. Although receiving treatment for chronic illnesses from more than one hospital is associated with poorer outcomes and delays in care [13, 14], current systems for cancer care, or at least colon cancer, may be robust to the causes of poorer survival associated with chronic illness care fragmented over different institutions.

On the other hand, total, inpatient, and outpatient costs of care were lower among patients whose transition from primary care to surgical cancer care did not involve a change in hospital. The more advanced the stage of colon cancer, the greater the savings. We observed cost differences in the six months following diagnosis, and this savings grew at twelve months following diagnosis, which may suggest the potential importance of continuity between primary care and cancer care during both initial treatment and on-going surveillance. Further, among stage III colon cancer patients, the fewer the hospitals involved in a patient’s primary, surgical, and medical oncologic care, the less expensive the total cost of care, echoing previous findings suggesting more integrated delivery of colon cancer care may reduce cost while preserving outcomes [9]. The extent to which these potential cost savings are driven by the improved management of comorbid conditions, reduction in the duplication of care, avoidance of preventable complications, or other factors remains unknown in our study. Others have found that greater primary care involvement in the year after colon cancer diagnosis is associated with fewer consultations with specialists, reduced radiographic studies, and fewer emergency room visits [23]. Electronic medical records and electronic referral systems are more likely to be shared among physicians part of the same hospital and have been shown to reduce duplication of services and improve communication between providers; however, their role has not been examined in cancer care [11, 48].

There are limitations to our study. First, despite the increasing integration of primary care provider networks with hospital systems [49], primary care is still a largely ambulatory enterprise, which may lead to errors in our assignment of a PCP’s primary hospital center. Second, a substantial proportion of patients could not be assigned to a PCP or did not undergo surgery; similar estimates of colon cancer patients without a PCP and/or not undergoing surgery have been reported by other investigators [30]. Understanding why such a significant number of patients do not seek regular primary care and do not receive surgical care for colon cancer remain important areas of study. Third, some patients classified as receiving primary care and surgical care at different hospitals may have in fact received care within a single integrated health system. As of 2013, 28% of registered U.S. hospitals were part of such an integrated network [50]. Failing to account for two different hospitals that are part of an integrated system would likely bias our findings towards the null. Fourth, we do not have data on gastroenterologist, who often make the diagnosis of colorectal cancer; however, previous research demonstrates the role of primary care on colorectal cancer outcomes is independent of gastroenterology care [30] and the number of visits with gastroenterologists in the first year of colorectal cancer diagnosis is negligible compared to the number of visits with PCPs [22]. Fifth, using claims data, we are unable to determine why patients and their referring PCPs may select particular surgeons or hospitals for cancer care. We address this analytically by adjusting for observable differences between patients, providers, and hospitals using propensity score methods; accounting for clustering of similar patients within hospitals; and restricting our sample only to hospitals that provide both primary and surgical care; however, unobservable cofounders may remain. Also, our volume measures are based on Medicare claims data only; however, volume measures determined from Medicare data are highly correlated with volume measures determined from all-payer data [51]. Finally, SEER-Medicare data only include claims for patients in fee-for-service Medicare; therefore, our findings may not be generalizable to younger patients—Medicare eligibility begins at age 65—or patients enrolled in preferred provider organizations, health maintenance organizations, or other types of health insurance programs.

Despite these limitations, this study suggests both the potential and challenges of policies that seek to better integrate primary care with cancer care. For example, the new oncology care model in the U.S. financially incentivizes patient care coordination across disciplines. It does not, however, address the systems challenges faced by providers delivering care or patients receiving care potentially fragmented across multiple institutions or systems. A large proportion of patients seek care from hospitals or systems outside their PCP’s and significant population of patients do not have a PCP prior to cancer diagnosis. Therefore, health systems must consider the optimal way to improve care delivery for these patients too, potentially relying on care delivery models that are equipped to span institutions and ease care transitions for patients receiving care from different providers, hospitals, and systems. At the same time, our findings suggest costs are lower among patients who receive primary care and cancer care from a single system, reinforcing the potential financial benefits of integrating the delivery of primary care and specialty care for cancer patients.

## Supporting Information

S1 Table Billing Codes used for the identification of colon cancer surgery. (DOCX)

S2 Table Balance of Covariates after propensity score matching. (DOCX)

S3 Table Sensitivity analyses for effect estimates in Table 2, for those receiving both primary and surgical care at the same hospital versus different hospitals. (DOCX)
