# Supplementary material for: The Value of Continuity between Primary Care and Surgical Care in Colon Cancer
Source: PLoS One. 2016 May 24;11(5):e0155789. doi: 10.1371/journal.pone.0155789 (PMC4878733; doi:10.1371/journal.pone.0155789)
Supplement: S1 Table — (DOCX) [file pone.0155789.s001.docx]

**S1 Table. Billing Codes used for the identification of colon cancer surgery.**

| **Type of Billing Code** | L**ist of specific codes** |
| --- | --- |
| **International Classification of Disease (ICD)-9 Codes** | 17.3-17.36, 17.39  45.7-45.76, 45.79  45.8-45.83 |
| **Current Procedural Terminology (CPT) Codes** | 44140, 44141, 44143-44147  44150-44158  44160  44204-44208, 44210 |
