# Supplementary material for: The Value of Continuity between Primary Care and Surgical Care in Colon Cancer
Source: PLoS One. 2016 May 24;11(5):e0155789. doi: 10.1371/journal.pone.0155789 (PMC4878733; doi:10.1371/journal.pone.0155789)
Supplement: S2 Table — (DOCX) [file pone.0155789.s002.docx]

**S2 Table. Balance of Covariates after propensity score matching.**

|  | **Primary and Surgical Care at the Same Hospital** | | **Primary and Surgical Care at Different Hospitals** | **Percent bias** |
| --- | --- | --- | --- | --- |
| **PATIENT-LEVEL PREDICTORS** | | | |  |
| **Age**  >65-70  71-75  76-80  81-85  >86 | 13.8%  22.1%  24.9%  21.9%  17.4% | | 13.4%  21.1%  24.4%  22.6%  18.4% | 1.1  2.3  1.0  -1.7  -2.7 |
| **Female** | 57.9% | | 58.6% | -1.5 |
| **Race**  White  Black  Other | 87.1%  6.8%  6.1% | | 87.9%  6.7%  5.4% | -2.4  0.6  2.7 |
| **Census Tract Income**  Lowest Quartile  2^nd^ Quartile  3^rd^ Quartile  Highest Quartile | 26.2%  24.6%  24.1%  25.1% | | 25.4%  24.6%  24.5%  25.5% | 1.8  0.0  -1.0  -0.8 |
| **Urban/Rural Residence**  ≥1 million population  ≥250000 to < 1 mil  <250,000 | 55.7%  28.1%  16.1% | | 54.7%  29.3%  16.0% | 2.1  -2.5  0.3 |
| **Charlson Comorbidity Score**  0  1  ≥2 | 53.7%  26.3%  20.0% | | 55.2%  25.6%  19.1% | -3.1  1.6  2.1 |
| **Tumor Grade**  Well Differentiated  Moderately Differentiated  Poorly Differentiated  Undifferentiated | 9.7%  69.3%  19.5%  1.5% | | 9.7%  69.5%  19.4%  1.4% | 0.2  -0.6  0.2  1.0 |
| **Adequate Lymph Node Resection** | 62.0% | | 62.8% | -1.6 |
| **PROVIDER-LEVEL CHARACTERISTICS** | | | |  |
| **Yearly surgical volume**  Lowest Quartile  2^nd^ Quartile  3^rd^ Quartile  Highest Quartile | 21.6%  19.8%  27.5%  31.1% | 20.3%  19.6%  29.1%  31.0% | | 3.2  0.4  -3.5  0.3 |
| **HOSPITAL-LEVEL CHARACTERISTICS** | | | |  |
| **Volume of Patients**  **Surgical Hospital**  Lowest Quartile  2^nd^ Quartile  3^rd^ Quartile  Highest Quartile | 25.0%  23.8%  25.9%  25.2% | 23.9%  24.5%  26.1%  25.6% | | 2.6  -1.4  -0.5  -0.7 |
| **NCI Status**  Surgical Hospital | 2.1% | 2.2% | | -0.3 |
| **Academic center**  Surgical Hospital  PCP Hospital | 52.0%  52.2% | 53.0%  53.0% | | -2.2  -1.6 |
| **For Profit Status**  Surgical Hospital  PCP Hospital | 8.4%  8.5% | 8.0%  8.5% | | 1.6  0.0 |
| **OVERALL DISTRIBUTION OF ALL COVARIATES** | | | |  |
| **Before Matching**  **After Matching** | --  -- | --  -- | | 5.5  1.4 |
