# Supplementary material for: The Value of Continuity between Primary Care and Surgical Care in Colon Cancer
Source: PLoS One. 2016 May 24;11(5):e0155789. doi: 10.1371/journal.pone.0155789 (PMC4878733; doi:10.1371/journal.pone.0155789)
Supplement: S3 Table — (DOCX) [file pone.0155789.s003.docx]

**S3 Table. Sensitivity analyses for effect estimates in Table 2, for those receiving both primary and surgical care at the same hospital versus different hospitals.**

|  | **Hazard Ratio**  **for All-Cause Mortality**  **(HR, 95%CI)** | **Subhazard Ratio for Colon Cancer Specific Mortality**  **(SHR, 95%CI)** | **Dollars Saved on Cost at 12 months**  **(Dollars, 95% CI)** |
| --- | --- | --- | --- |
| **Estimates from Table 3** | 1.04 (0.99-1.09) | 1.02 (0.97-1.06) | $8836  ($2,746-$14,577) |
| **Including patients with missing data, using multiple imputation** | 0.97 (0.94-1.03) | 1.01 (0.97-1.05) | $9234  ($4,987-$16,021) |
| **Adding substage in adjustment model**^†^ | 1.01 (0.96-1.03) | 1.03 (0.95-1.09) | $8,763  ($3,291-$13,765) |
| **Propensity score-matching using only patient characteristics** | 0.99 (0.96-1.03) | 0.97 (0.93-1.02) | $9099  ($3,111-$17,275) |
| **Cost Sensitivity Analyses** | | | |
| **Cost of care at 12 months, amongst only those surviving to 12 months** | N/A | N/A | $7418  ($1638-$11,850) |
| **Cost of care at 6 months** | N/A | N/A | $3648  ($718-$7,006) |
| **Cost of care at 6 months, amongst only those surviving to 6 months** | N/A | N/A | $4178  ($850-$6,940) |
| **Sensitivity Analyses for Stage III Colon Cancer Patients:**  **Number of Different Hospitals where Patient Received**  **Primary Care, Surgical Care, & Medical Oncologic Care**^‡^ | | | |
| 1 Hospital  2 Hospitals  3 Hospitals | 0.95 (0.91-1.04)  0.96 (0.91-1.05)  Ref | 0.95 (0.92-1.03)  0.95 (0.92-1.07)  Ref | $25,973  ($20,843-$31,114)  $19,297  ($16,013-$24,765)  Ref |

*: Sensitivity analyses for effect estimates in Table 2. All estimates are from propensity score-matched doubly robust models. Estimates are fully adjusted for all patient, provider, and hospital characteristics listed in Table 1 (except substage unless otherwise noted)

†: Sample restricted to those diagnosed between 2004 onward, for whom this data is available

‡: Adjusted logistic regression only, not propensity score matched given three comparison groups
